# Anthropometric parameters and their associations with cardio-metabolic risk in Chinese working population

**DOI:** 10.1186/s13098-015-0032-5

**Published:** 2015-04-24

**Authors:** Xiaojun Ouyang, Qinlin Lou, Liubao Gu, Gary T Ko, Yongzhen Mo, Haidi Wu, Rongwen Bian

**Affiliations:** Diabetes Care and Research Center, Jiangsu Province Institute of Geriatrics, 30 Luojia Road, Nanjing, 210024 China; Department of Endocrinology and Metabolism, Jiangsu Province Institute of Geriatrics, 30 Luojia Road, Nanjing, 210024 China; Department of Medicine and Therapeutics, Prince of Wales Hospital, the Chinese University of Hong Kong, Hong Kong, China

**Keywords:** Body mass index, Waist circumference, Waist-to-hip ratio, Obesity, Cardio-metabolic risks

## Abstract

**Background:**

There remains controversy regarding which of the anthropometric indicators best defines obesity. In this study, we compared the efficacy of using body mass index (BMI), waist circumference (WC) and waist-to-hip ratio (WHR) in the diagnosis of obesity and assessed their associations with diabetes, hypertension, and dyslipidemia in an urban working population in China.

**Methods:**

Anthropometric measurements, blood pressure, plasma lipids, fasting and 2-hour plasma glucose (PG) levels by a 75 gram oral glucose tolerance test (OGTT) were obtained from 2603 working Chinese who had no history of cardiovascular diseases or diabetes. Cardio-metabolic risk factors including high blood pressure, dyslipidemia, and glucose intolerance were evaluated. The diagnoses of overweight and obesity were based on the WHO definitions with BMI for general obesity and WC and WHR for central obesity.

**Results:**

Based on BMI, WC and WHR, there were 31.3%, 16.6%, 35.2% of the studied subjects, respectively, being overweight and 2.0%, 5.6%, 9.2% being obese. Among women but not men, more overweight and obese subjects were diagnosed using WHR and WC. The number of cardio-metabolic risks was higher by WC criterion than BMI and WHR in the whole group (*p* <0.05) and female subjects (*p* <0.01). Comparing the three anthropometric indexes predicting hypertension, hyperglycemia, dyslipidemia and multiple cardio-metabolic risks, for women, it was WC having the largest areas under ROC curves (0.759, 0.746, 0.701 and 0.773 respectively); while in men, it was WC for hypertension, WHR for hyperglycemia, BMI for dyslipidemia and WC for multiple cardio-metabolic risks (areas under ROC curves were 0.658, 0.686, 0.618 and 0.695 respectively).

**Conclusions:**

Among Chinese working population, the need of lower cutoff values to define overweight and obesity were observed. Central obesity indicator (WC) is the preferred measure to predict the presence of cardio-metabolic risk in Chinese female subjects.

## Introduction

The increasing prevalence of obesity is a major public health problem worldwide. Wu *et al.* showed that there were 14.7% of Chinese being overweight (body mass index, BMI 25.0–29.9 kg/m^2^) and another 2.6% being obese (BMI ≥30.0 kg/m^2^) [1]. Obesity is a major independent risk factor for metabolic disorders, such as diabetes, hypertension, insulin resistance and dyslipidemia. Their adverse effects on physical and social functioning and quality of life were also well documented [2,3].

Recently, many studies had been using different definitions to classify obesity for Chinese, such as BMI ≥25 to <27.5 kg/m^2^ as overweight and a BMI ≥27.5 kg/m^2^ as general obesity [4], and a waist circumference (WC) ≥90 cm in men or WC ≥80 cm in women as abdominal obesity [5], while local experts in Mainland China adopted a BMI ≥24 to <28 kg/m^2^ as overweight and BMI ≥28 kg/m^2^ as general obesity, and WC ≥85 cm for men and ≥80 cm for women as abdominal obesity [6].

To delineate obesity, various anthropometric measures have been proposed such as BMI, WC and waist-to-hip ratio (WHR). However, controversy still exists regarding which of these anthropometric indicators best defines obesity. Their relationships with cardio-metabolic risk also deserve further exploration. The aim of the present study is to compare the efficacy of BMI, WC and WHR in the diagnosis of obesity in a Chinese working population. We also attempted to assess their respective associations with diabetes, hypertension, and dyslipidemia by gender.

## Research design and methods

We recruited 2603 subjects in Nanjing, an urban city of China with a population of 5.5 million. The study was conducted in the period between October 2003 and March 2005 (details of study procedure had been published previously) [7]. The survey was approved by Jiangsu Province Health Administrative Department and Ethics Committee. Every subject conxsented to participate in the study.

We measured body height, body weight, WC, Hip circumference with subjects in light clothing without shoes. Fat mass (kg) and body fat percent (BF%) were assessed with bioelectrical impedance equipment (TANITA, TBF-300, France). The equation for BF% used by this model of Tanita was developed by Brozek [8]:

$$ \mathrm{B}\mathrm{F}\%={\left(4.57/\mathrm{body}\ \mathrm{density}-4.142\right)}^{\ast }100 $$

Venous blood was collected from the ante-cubital vein after a 12-hour overnight fast. A 75 gram anhydrous glucose load oral glucose tolerance test (OGTT) was performed after fasting blood samples were collected. Blood for glucose assay was containing sodium fluoride, and fasting plasma glucose (FPG) and 2-hour plasma glucose (2hPG) levels were determined by the glucose–oxidase method. Serum total cholesterol (TC), high-density lipoprotein cholesterol (HDL-C), and triglyceride (TG) concentrations were determined enzymatically. Low-density lipoprotein cholesterol (LDL-C) was calculated using the Friedewald’s formula [9]. Serum fasting insulin (FINS) and 2-hour post-OGTT insulin (2hINS) levels were measured by radioimmunoassay (RIA) with BNIBT kits (North Institute of Biological Technology, Beijing, China), of which the intra-assay coefficient of variation was below 10% and the inter-assay coefficient of variation was below 15%.

Homeostasis model assessment of insulin resistance (HOMA-IR), an index which represents insulin resistance, was calculated according to the following formula [10]:

$$ \mathrm{HOMA}\hbox{-} \mathrm{I}\mathrm{R}=\left[\mathrm{FINS}\ \left(\upmu \mathrm{U}/\mathrm{ml}\right)\times \mathrm{F}\mathrm{P}\mathrm{G}\ \left(\mathrm{mmol}/\mathrm{L}\right)\right]/22.5 $$

High HOMA-IR was defined as HOMA-IR ≥2.69 [11,12].

The homeostasis model assessment of β-cell function (HOMA-β) was used to evaluate basal insulin secretion. This was calculated as:

$$ \mathrm{HOMA}\hbox{-} \upbeta = \left[\mathrm{F}\mathrm{INS}\left(\upmu \mathrm{U}/\mathrm{ml}\right) \times 20\right]/\left[\mathrm{F}\mathrm{P}\mathrm{G}\left(\mathrm{mmol}/\mathrm{L}\right)\hbox{-} 3.5\right] $$

Low HOMA-β was defined as HOMA-β ≤111.2 [13].

### Definitions

Hypertension was defined as systolic and/or diastolic blood pressure (BP) ≥140/90 mmHg and/or current use of antihypertensive agents. Hyperglycemia was defined as FPG ≥5.6 mmol/L and/or 2hPG ≥7.8 mmol/L or with known diabetes history. Dyslipidmia was defined as having 1 or more of the following: TC ≥5.18 mmol/L, HDL-C <1.04 mmol/L, LDL-C ≥3.37 mmol/L and TG ≥1.70 mmol/L [14]. The presence of 2 or more of the above three cardio-metabolic risk factors (i.e. hypertension, hyperglycemia, dyslipidemia) was defined as having “multiple metabolic risks”.

The diagnoses of overweight and obese were based on World Health Organization (WHO) classification [15]. Those with a BMI of 25.0–29.9 kg/m^2^ were classified as overweight, whilst those with a BMI ≥30.0 kg/m^2^ were classified as obese. Men with a WC of 94–101.9 cm and women of 80–87.9 cm were classified as overweight, whilst men with a WC ≥102.0 cm and women ≥88.0 cm were classified as obese. Men with a WHR of 0.90–0.99 and women of 0.80–0.84 were classified as overweight, whilst men and women with a WHR ≥1.00 and ≥0.85 were classified as obese, respectively [15].

### Statistical analysis

Statistical analysis was performed using the SPSS (version 15.0) software for Windows. Continuous variables were presented as mean ± SD and categorical data as number (%). Difference in means between groups was tested using one-way analysis of variance (ANOVA) and Chi-square (χ^2^) test where appropriate. A *p*-value <0.05 (two-tailed) was considered to be statistically significant. The univariate analysis and logistic regression analysis were conducted to compare the differences among the 3 anthropometric indexes in their relationships with hypertension, hyperglycemia and dyslipidemia. Receiver operating characteristic (ROC) curve analyses and area-under-curve (AUC) were plotted and compared with regard to their associations with various metabolic risk factors, and were used to determine the optimal values for BMI, WC and WHR in diagnosing obesity by gender. The maximal Youden Index (YI), defined as the sum of specificity and sensitivity minus one and its associated optimal cutoff point on the ROC curves was also estimated.

## Results

Of the 2603 subjects, 1590 (61.1%) were men and 1013 (38.9%) were women. Their mean age was 47.3 ± 11.6 years (median 46 years, range 23 to 79 years). Compared to women, men had higher BMI, WC, WHR, BP, PG, TG, insulin levels, HOMA-IR, and more smokers, hypertension, hyperglycemia and dyslipidemia, but lower HDL-C level, fat mass and BF% (Table 1). There were 42% subjects having a high HOMA-IR level and one quarter of the whole study group had a low HOMA-β level. Among the 1013 women, 365 (36.0%) were menopausal. As compared to pre-menopausal women, those with menopause had worse cardio-metabolic profiles (higher anthropometric parameters, fat mass, BF%, BP, lipid profiles, glycemic indexes, higher Homa-IR and lower Homa-β) (detailed data not shown).

**Table 1:** **Clinical and biochemical characteristics of the 2603 Chinese subjects**

	**Total (n = 2603)**	**Men (n = 1590)**	**Women (n = 1013)**	***p*** **-value** ^*^
Age (years)	47.3 ± 11.6	47.6 ± 11.6	47.2 ± 11.6	0.373
BMI (kg/m^2^)	23.7 ± 2.9	24.4 ± 2.6	22.6 ± 3.0	0.000
WC (cm)	82.5 ± 9.2	86.3 ± 7.3	76.4 ± 8.6	0.000
WHR	0.85 ± 0.06	0.89 ± 0.05	0.81 ± 0.05	0.000
Fat mass (kg)	26.1 ± 6.6	24.5 ± 11.6	28.5 ± 7.3	0.000
BF%	18.0 ± 5.7	17.7 ± 5.8	18.2 ± 5.6	0.044
SBP (mmHg)	119.9 ± 16.2	122.8 ± 15.5	115.4 ± 16.3	0.000
DBP (mmHg)	78.0 ± 10.1	80.3 ± 9.9	74.4 ± 9.3	0.000
TC (mmol/L)	4.99 ± 0.86	4.98 ± 0.84	5.02 ± 0.89	0.213
HDL-C (mmol/L)	1.41 ± 0.29	1.35 ± 0.29	1.49 ± 0.28	0.000
LDL-C (mmol/L)	2.96 ± 0.68	2.95 ± 0.67	2.98 ± 0.70	0.239
TG (mmol/L)	1.60 ± 1.11	1.82 ± 1.17	1.24 ± 0.89	0.000
Fasting PG (mmol/L)	5.0 ± 0.9	5.1 ± 1.0	4.9 ± 0.8	0.000
OGTT 2 hr PG (mmol/L)	6.4 ± 2.4	6.6 ± 2.5	6.3 ± 2.2	0.003
Fasting serum Insulin (uIU/ml)	12.7 ± 7.1	13.0 ± 7.3	12.3 ± 6.9	0.010
OGTT 2 hr serum Insulin (uIU/ml)	64.2 ± 49.1	65.9 ± 49.2	61.6 ± 48.6	0.030
HOMA-IR	2.85 ± 1.85	2.98 ± 1.97	2.71 ± 1.76	0.000
High HOMA-IR (%)	42.0	45.3	36.9	0.004
Homa-β	204.1 ± 177.0	202.1 ± 187.5	206.1 ± 157.3	0.575
Low HOMA-β (%)	25.3	27.1	22.5	0.009
Smoking (%)	31.5	50.2	2.0	0.000
Hypertension (%)	30.9	35.8	23.4	0.000
Newly diagnosed (%)	7.8	9.8	4.5	0.000
Known hypertensive (%)	23.1	26.0	18.9	0.000
Hyperglycemia (%)	19.3	25.1	19.5	0.001
Isolated IFG	1.2	1.7	0.6	0.012
Isolated IGT	10.1	10.1	10.1	1.0
Combined IFG/IGT	2.1	2.7	1.3	0.013
Diabetes mellitus	5.9	6.5	4.8	0.000
Dyslipidemia (%)	59.6	65.6	50.0	0.000
TC ≥5.18 mmol/L	40.2	39.5	41.3	0.369
HDL-C <1.04 mmol/L	9.0	12.3	3.9	0.000
LDL-C ≥3.37 mmol/L	25.5	24.8	26.8	0.271
TG ≥1.70 mmol/L	32.2	41.6	17.2	0.000

*p-value:comparing men and women.

BMI, body mass index; WC, waist circumference; WHR, waist-to-hip ratio; BF%, body fat percent; SBP, systolic blood pressure; DBP, diastolic blood pressure; TC, total cholesterol; HDL-C and LDL-C, high- and low-density lipoprotein cholesterol; TG, triglyceride; OGTT, oral glucose tolerance test; PG, plasma glucose; IFG, impaired fasting glucose; IGT, impaired glucose tolerance; high HOMA-IR = HOMA-IR ≥2.69; low HOMA-β = HOMA-β ≤111.2; isolated IFG = fasting PG ≥6.1 and <7.0 mmol/L and 2 h PG <7.8 mmol/L; isolated IGT = fasting PG <6.1 mmol/L and 2 h PG ≥7.8 and <11.1 mmol/L; combined IFG/IGT = fasting PG ≥6.1 and <7.0 mmol/L and/or 2 h PG ≥7.8 and <11.1 mmol/L; diabetes mellitus = fasting PG ≥7.0 mmol/L and/or 2 h PG ≥11.1 mmol/L.

According to BMI, WC and WHR criteria respectively, 31.3% (n = 816), 16.6% (431), 35.2% (917) subjects had overweight and 2.0% (52), 5.6% (147), 9.2% (239) subjects had obesity. Among women but not men, more overweight and obese subjects were diagnosed by WHR and WC. In the obese group, 78.2% and 94.1% were women by WC and WHR criteria, but only 38.5% when using BMI.

Using WC criteria, both obese men and women had about 2 cardio-metabolic risk factors. The number of cardio-metabolic risk factors was higher by WC criteria than BMI and WHR in total (1.99 ± 0.93 vs. 1.85 ± 0.85 vs. 1.62 ± 0.98, respectively, *p* <0.05) and female subjects (1.99 ± 0.93 vs. 1.95 ± 0.76 vs. 1.61 ± 0.97, respectively, *p* <0.01), but no difference was found in male subjects or between BMI and WHR criteria (2.0 ± 0.95 vs. 1.78 ± 0.91 vs. 1.86 ± 1.10, respectively, *p*-value: NS).

The percentage of subjects having cardio-metabolic risk factors by the three anthropometric measures increased as with obesity worsened. According to BMI, WC and WHR criteria, 23.4%, 26.3% and 23.6% normal subjects as well as 63.5%, 67.3% and 51.9% obese subjects had multiple (2 or more) cardio-metabolic risk factors, respectively.

With logistic regression analysis, the odd-ratio (OR) of hypertension, hyperglycemia, and dyslipidemia increased as the anthropometric parameters increased (*p*-values all <0.01). After adjustment for age and smoking in obese women, WC had the highest OR in predicting hypertension and hyperglycemia, while WHR had the highest OR to predict dyslipidemia.. Among obese men, WC for hypertension and WHR for hyperglycemia had the highest OR (Table 2). All 3 anthropometric parameters lost their statistical significance in predicting dyslipidemia in obese male subjects. But among overweight men, WC had the highest OR.

**Table 2:** **Multiple logistic regression analysis of the associations between BMI, WC, WHR and cardio-metabolic risks**

**OR (95% CI)**		**Hypertension**			**Hyperglycemia**			**Dyslipidemia**		
		**Crude**	**Adjusted for age**	**Adjusted for age and smoking**	**Crude**	**Adjusted for age**	**Adjusted for age and smoking**	**Crude**	**Adjusted for age**	**Adjusted for age and smoking**
Men (n = 1590)										
BMI	overweight	* 1.86 (1.46-2.35)	* 2.15 (1.67-2.77)	* 2.62 (2.08-3.30)	* 1.42 (1.07-1.89)	* 1.56 (1.16-2.09)	* 2.15 (1.65-2.79)	* 1.55 (1.21-1.98)	* 1.54 (1.21-1.97)	* 1.82 (1.45-2.28)
	obese	0.78 (0.32-1.90)	0.93 (0.37-2.37)	1.72 (0.78-3.80)	* 2.77 (1.16-6.63)	* 3.41 (1.40-8.31)	* 6.33 (2.92-13.72)	1.99 (0.73-5.47)	1.99 (0.72-5.47)	2.26 (0.96-5.30)
WC	overweight	1.28 (0.91-1.79)	1.14 (0.80-1.62)	* 2.01 (1.48-2.72)	1.18 (0.82-1.71)	1.08 (0.74-1.57)	* 2.32 (1.69-3.20)	1.27 (0.87-1.87)	1.27 (0.87-1.87)	* 1.94 (1.38-2.71)
	obese	* 3.04 (1.22-7.57)	2.88 (0.94-5.68)	* 4.20 (1.86-9.47)	* 2.44 (1.04-5.76)	1.84 (0.76-4.47)	* 6.30 (2.89-13.70)	1.08 (0.41-2.84)	1.08 (0.41-2.84)	1.88 (0.83-4.25)
WHR	overweight	* 1.48 (1.16-1.88)	* 1.46 (1.13-1.81)	* 2.01 (1.60-2.53)	* 2.31 (1.74-3.07)	* 2.33 (1.75-3.11)	* 2.97 (2.28-3.86)	* 1.31 (1.03-1.68)	* 1.31 (1.02-1.68)	* 1.64 (1.31-2.05)
	obese	2.38 (0.72-7.91)	2.19 (0.62-7.77)	2.81 (0.92-8.57)	* 6.68 (2.03-22.00)	* 6.48 (1.93-21.75)	* 8.09 (2.68-24.37)	1.02 (0.30-3.50)	1.02 (0.30-3.50)	1.50 (0.47-4.85)
Women (n = 1013)										
BMI	overweight	1.32 (0.84-2.10)	* 1.99 (1.20-3.29)	* 3.12 (2.14-4.14)	0.73 (0.43-1.24)	0.97 (0.55-1.70)	* 1.87 (1.25-2.81)	0.98 (0.57-1.67)	1.07 (0.60-1.92)	1.38 (0.88-2.16)
	obese	1.33 (0.40-4.42)	2.26 (0.58-8.83)	* 3.49 (1.16-10.50)	1.15 (0.34-3.96)	1.70 (0.45-6.40)	* 3.15 (1.07-9.26)	1.88 (0.22-15.97)	3.47 (0.34-34.96)	1.86 (0.34-10.55)
WC	overweight	* 2.79 (1.74-4.47)	1.37 (0.82-2.28)	* 1.92 (1.28-2.88)	* 2.27 (1.33-3.88)	1.27 (0.73-2.23)	* 1.71 (1.09-2.70)	* 2.51 (1.48-4.23)	1.39 (0.77-2.52)	1.53 (0.98-2.40)
	obese	* 7.24 (3.67-14.31)	* 2.17 (1.03-4.60)	* 3.95 (2.35-6.63)	* 6.86 (3.29-14.31)	* 6.86 (1.17-5.62)	* 3.34 (1.97-5.68)	* 3.38 (1.42-8.03)	1.07 (0.39-2.93)	1.48 (0.74-2.97)
WHR	overweight	1.19 (0.80-1.77)	0.96 (0.63-1.48)	1.16 (0.76-1.76)	1.10 (0.69-1.78)	0.95 (0.58-1.55)	0.88 (0.54-1.44)	* 1.47 (1.06-2.04)	1.10 (0.77-1.59)	1.20 (0.84-1.72)
	obese	1.18 (0.72-1.95)	1.10 (0.66-1.85)	* 2.07 (1.36-3.17)	* 1.94 (1.13-3.33)	* 1.91 (1.12-3.27)	* 2.44 (1.53-3.87)	* 1.82 (1.09-3.06)	1.38 (0.76-2.52)	* 1.64 (1.03-2.64)

Normal as reference (OR = 1), * p-value = statistically significant with p <0.05.

CI, confidence interval; OR, odds ratio; BMI, body mass index; WC, waist circumference; WHR, waist-to-hip ratio.

Table 3 shows the ROC curves to determine the appropriate BMI, WC and WHR values for detecting the presence of various metabolic risks in males and females respectively. The cutoff value of BMI to predict multiple metabolic risks were 24.6 kg/m^2^ and 22.6 kg/m^2^ in man and women, respectively. Those of WC and WHR were 85.5 cm and 77.5 cm, 0.89 and 0.83 in men and women, respectively. In women, the largest AUC were detected by WC (respectively 0.759, 0.746, 0.701 and 0.773 for hypertension, hyperglycemia, dyslipidemia and multiple metabolic risks). While in men, the corresponding figures were by WC for hypertension, WHR for hyperglycemia, BMI for dyslipidemia and WC for multiple metabolic risks (AUC = 0.658, 0.686, 0.618 and 0.695 respectively with their 95% CIs overlapping with each other).

**Table 3:** **The cutoff values, sensitivity and specificity for cardio-metabolic risks by BMI, WC and WHR**

	**BMI (kg/m** ^**2**^ **)**				**WC (cm)**				**WHR**			
	**Cutoff**	**Se (%)**	**Sp (%)**	**YI**	**Cutoff**	**Se (%)**	**Sp (%)**	**YI**	**Cutoff**	**Se (%)**	**Sp (%)**	**YI**
Men (n = 1590)												
Hypertension	24.2	67.3	54.8	0.22	85.5	69.2	53.9	0.23	0.89	59.4	59.8	0.19
Hyperglycemia	24.9	57.5	61.2	0.19	88.5	55.3	68.4	0.24	0.91	55.9	73	0.29
Dyslipidemia	24.0	62.5	54.2	0.17	87.5	46.5	68.1	0.15	0.88	60.6	56.7	0.17
Multiple risk factors	24.6	63.8	63.0	0.27	85.5	72.8	55.6	0.28	0.89	63.4	62.8	0.26
Women (n = 1013)												
Hypertension	22.9	69.9	66.8	0.37	77.5	67.7	70.2	0.38	0.82	65.0	66.8	0.32
Hyperglycemia	23.6	54.9	70.3	0.25	77.5	67.3	67.1	0.34	0.84	53.7	80.4	0.34
Dyslipidemia	22.2	58.7	68.6	0.27	75.5	58.7	74.2	0.33	0.81	55.5	67.8	0.23
Multiple risk factors	22.6	71.4	63.7	0.35	77.5	67.7	72.4	0.40	0.83	63.6	72.7	0.36

BMI, body mass index; WC, waist circumference; WHR, waist-to-hip ratio, Se: Sensitivity, Sp: Specificity, YI: Youden Index.

## Discussion

To ensure comparability with other studies, our study selected the criteria recommended by WHO classifications [4]. In this study, the prevalence of overweight/ obesity was 31.3%/ 2.0%, 16.6%/ 5.6%, 35.2%/ 9.2% by BMI, WC and WHR respectively. These results exhibited lower prevalence of overweight and obesity than those seen in Western countries [16,17].

In agreement with other South Asian and Chinese data [18,19], our findings confirmed that quite a few subjects with normal weight were already associated with many cardio-metabolic risk factors and those with overweight or obesity, their associated risks were even higher. In other words, using a lower obesity cutoff value for Chinese population appears to be reasonable in a general health prevention point of view [20]. Nguyen *et al.* had suggested that the optimal BMI cutoff value being 23–24 kg/m^2^ for Chinese [21]. In the present study with Chinese working population, the optimal BMI cutoff values to detect the presence of multiple metabolic risk factors were 24.6 kg/m^2^ for men and 22.6 kg/m^2^ for women, while the WC cutoff values were 85.5 cm for men and 77.5 cm for women. All these values were closely approximate to those being reported by many Japanese and Korean studies [22,23], while slightly higher than those by studies in Hong Kong [24]. Actually, in the last international joint statement of diagnosing metabolic syndrome published in 2009, a population- and country-specific definition for elevated WC was highlighted [25]. Among Chinese and Asian, a WC cutoff of 85–90 cm for men and 80 cm for women were suggested to define abdominal obesity. These figures were similar to what we have found in this study.

More women were diagnosed using central obesity indexes (WC and WHR) than BMI, while men had similar obese prevalence by all 3 measures. Interestingly, we also found most centrally obese subjects were women. Similar findings were also obtained from a study in Iran that the prevalence of abdominal obesity was much higher in women (53.5%) than in men (12.5%) [26]. Hauner *et al.* also reported that women more often had an increased WC as compared to men [27]. All these findings suggested the WC and WHR criteria for central obesity in men may be too cautiously high. In accord to this, based on HOMA-IR level correlating to metabolic parameters among Japanese subjects, Kamezaki F *et al.* demonstrated that WC cutoff level should be reduced and more strictly managed [28].

It is well documented that WC more closely correlates with the abdominal visceral fat than either WHR or BMI. In agreement with our findings, most cross-sectional studies showed a stronger association of cardiovascular risk factors with central obesity (based on WC or WHR) than with general obesity (BMI) in Chinese as well as other ethnic populations [29-31]. The prospective AusDiab Study confirmed that abdominal obesity was associated with ORs between 2 and 5 for incident type 2 diabetes, dyslipidemia and hypertension at 5-year follow up [32].

Insulin resistance contributed largely to the underlying pathogenesis of hyperglycemia, hypertension and dyslipidemia, while central obesity has been closely linked to insulin resistance. Among Chinese teenagers, Yin *et al.* have shown that those with 3 or more cardiovascular risk factors or metabolic abnormalities as compared to those without risk factors had a 4-fold increase in HOMA-IR and a mean difference in WC of 23 cm [33]. Among Canadians of different ethnicities, Chateau-Degat *et al.* have also demonstrated that those in the highest WC quartile had the highest HOMA-IR highlighting the importance of increased abdominal obesity on metabolic parameters [34]. Interestingly, they also showed that WC did not have a similar deleterious impact according to ethnicity suggesting the need for an ethnic-based definition.

Yet, some studies did not show consistently significant differences between measures of general and central obesity [35-37]. In the Decoda study, though diabetes had stronger association with waist-to-stature ratio (WSR) than BMI (*p* = 0.001) in men and a similar higher risk with WC and WSR than BMI (both *p* <0.05) in women, they also found that hypertension had stronger association with BMI than WHR in men (*p* <0.001) and a highest risk with BMI than other parameters in women [38]. It is now widely accepted that, on the one hand, abdominal obesity is a main predictive factor of cardio-metabolic risk, while on the other hand, BMI, WC and WHR are all useful tools for assessing adiposity in clinical practice [39,40].

The present study has its own limitations. This is a cross-sectional epidemiological study with surrogate measures of general and central obesity instead of direct measure of body composition being used for analysis in the prediction of metabolic risks. In addition, our subjects came from a working population who might not be able to represent all the socioeconomic status of the general population. The biased nature of our sample was also expressed with more than 40% of our subjects having a high HOMA-IR level and up to a quarter of them having a low HOMA-β level, suggesting a less favorable metabolic indices among our study subjects. In accord to this, high risk for cardiovascular disease and suboptimal health among working population has been reported both in Chinese and Caucasians [41,42].

## Conclusion

In this study, the number of cardio-metabolic risk factors increased with the degree of obesity in our Chinese working population. The need for a lower cutoff values to define overweight and obesity were observed. Men have a higher prevalence of overweight or general obesity than women, whereas women have a higher prevalence of abdominal obesity than men. WC, being the most important central obesity indicator, is the preferred measure to predict the presence of cardio-metabolic risk in Chinese women. WC also provides important information in Chinese men on their cardio-metabolic risk while BMI supplements significantly.

## Abbreviations

BMI: Body mass index
WC: Waist circumference
WHR: Waist-to-hip ratio
BP: Blood pressure
BF%: Body fat percent (BF%)
OGTT: Oral glucose tolerance test
FPG: Fasting plasma glucose
2hPG: 2-hour plasma glucose
TC: Total cholesterol
HDL-C: High-density lipoprotein cholesterol
TG: Triglyceride
LDL-C: Low-density lipoprotein cholesterol
FINS: Fasting insulin
2hINS: 2-hour post-OGTT insulin
RIA: Radioimmunoassay
HOMA-IR: Homeostasis model assessment of insulin resistance
HOMA-β: Homeostasis model assessment of β-cell function
WHO: World Health Organization
ANOVA: One-way analysis of variance
ROC: Receiver operating characteristic
AUC: Area-under-curve
OR: Odd-ratio
WSR: Waist-to-stature ratio
